# Supplementary material for: HIVprotI: an integrated web based platform for prediction and design of HIV proteins inhibitors
Source: J Cheminform. 2018 Mar 9;10:12. doi: 10.1186/s13321-018-0266-y (PMC5845081; doi:10.1186/s13321-018-0266-y)
Supplement: Supplementary file 2 — Additional file 2. Source code of HIVProtI web server. [file 13321_2018_266_MOESM2_ESM.zip › HIVprotI_Source-code/hivprotI/weka-3-6-6/doc/weka/core/TechnicalInformation.Type.html]

TechnicalInformation.Type


---


|  |  |  |
| --- | --- | --- |
| **Overview** \| **Package** \| **Class** \| **Tree** \| **Deprecated** \| **Index** \| **Help** \| **Weka's home** | |  |
| **PREV CLASS**   **NEXT CLASS** | **FRAMES**    **NO FRAMES**     **All Classes** |  |
| SUMMARY: NESTED \| ENUM CONSTANTS \| FIELD \| METHOD | DETAIL: ENUM CONSTANTS \| FIELD \| METHOD |  |


---


## weka.core Enum TechnicalInformation.Type

```
java.lang.Object
  java.lang.Enum<TechnicalInformation.Type>
      weka.core.TechnicalInformation.Type
```

**All Implemented Interfaces:**: java.io.Serializable, java.lang.Comparable<TechnicalInformation.Type>

**Enclosing class:**: TechnicalInformation

---

``` public static enum TechnicalInformation.Type extends java.lang.Enum<TechnicalInformation.Type> ```

the different types of information

---

**Enum Constant Summary**

`ARTICLE`
  
          An article from a journal or magazine.

`BOOK`
  
          A book with an explicit publisher.

`BOOKLET`
  
          A work that is printed and bound, but without a named publisher or sponsoring institution.

`CONFERENCE`
  
          The same as inproceedings.

`INBOOK`
  
          A part of a book, which may be a chapter (or section or whatever) and/or a range of pages.

`INCOLLECTION`
  
          A part of a book having its own title.

`INPROCEEDINGS`
  
          An article in a conference proceedings.

`MANUAL`
  
          Technical documentation.

`MASTERSTHESIS`
  
          A Master's thesis.

`MISC`
  
          Use this type when nothing else fits.

`PHDTHESIS`
  
          A PhD thesis.

`PROCEEDINGS`
  
          The proceedings of a conference.

`TECHREPORT`
  
          A report published by a school or other institution, usually numbered within a series.

`UNPUBLISHED`
  
          A document having an author and title, but not formally published.


| **Method Summary** | |
| --- | --- |
| `java.lang.String` | `getComment()`             returns the comment string |
| `java.lang.String` | `getDisplay()`             returns the display string |
| `java.lang.String` | `toString()`             returns the display string of the Type |
| `static TechnicalInformation.Type` | `valueOf(java.lang.String name)`             Returns the enum constant of this type with the specified name. |
| `static TechnicalInformation.Type[]` | `values()`             Returns an array containing the constants of this enum type, in the order they are declared. |

**Methods inherited from class java.lang.Enum**

`compareTo, equals, getDeclaringClass, hashCode, name, ordinal, valueOf`

**Methods inherited from class java.lang.Object**

`getClass, notify, notifyAll, wait, wait, wait`

**Enum Constant Detail**

### ARTICLE

```
public static final TechnicalInformation.Type ARTICLE
```

:   An article from a journal or magazine.

---


### BOOK

```
public static final TechnicalInformation.Type BOOK
```

:   A book with an explicit publisher.

---


### BOOKLET

```
public static final TechnicalInformation.Type BOOKLET
```

:   A work that is printed and bound, but without a named publisher or sponsoring institution.

---


### CONFERENCE

```
public static final TechnicalInformation.Type CONFERENCE
```

:   The same as inproceedings.

---


### INBOOK

```
public static final TechnicalInformation.Type INBOOK
```

:   A part of a book, which may be a chapter (or section or whatever) and/or a range of pages.

---


### INCOLLECTION

```
public static final TechnicalInformation.Type INCOLLECTION
```

:   A part of a book having its own title.

---


### INPROCEEDINGS

```
public static final TechnicalInformation.Type INPROCEEDINGS
```

:   An article in a conference proceedings.

---


### MANUAL

```
public static final TechnicalInformation.Type MANUAL
```

:   Technical documentation.

---


### MASTERSTHESIS

```
public static final TechnicalInformation.Type MASTERSTHESIS
```

:   A Master's thesis.

---


### MISC

```
public static final TechnicalInformation.Type MISC
```

:   Use this type when nothing else fits.

---


### PHDTHESIS

```
public static final TechnicalInformation.Type PHDTHESIS
```

:   A PhD thesis.

---


### PROCEEDINGS

```
public static final TechnicalInformation.Type PROCEEDINGS
```

:   The proceedings of a conference.

---


### TECHREPORT

```
public static final TechnicalInformation.Type TECHREPORT
```

:   A report published by a school or other institution, usually numbered within a series.

---


### UNPUBLISHED

```
public static final TechnicalInformation.Type UNPUBLISHED
```

:   A document having an author and title, but not formally published.


**Method Detail**

### values

```
public static TechnicalInformation.Type[] values()
```

:   Returns an array containing the constants of this enum type, in
    the order they are declared. This method may be used to iterate
    over the constants as follows:

    ```
    for (TechnicalInformation.Type c : TechnicalInformation.Type.values())
        System.out.println(c);
    ```

    :   **Returns:**: an array containing the constants of this enum type, in the order they are declared

---


### valueOf

```
public static TechnicalInformation.Type valueOf(java.lang.String name)
```

:   Returns the enum constant of this type with the specified name.
    The string must match *exactly* an identifier used to declare an
    enum constant in this type. (Extraneous whitespace characters are
    not permitted.)

    :   **Parameters:**: `name` - the name of the enum constant to be returned. **Returns:**: the enum constant with the specified name **Throws:**: `java.lang.IllegalArgumentException` - if this enum type has no constant with the specified name: `java.lang.NullPointerException` - if the argument is null

---


### getDisplay

```
public java.lang.String getDisplay()
```

:   returns the display string

    :   **Returns:**: the display string

---


### getComment

```
public java.lang.String getComment()
```

:   returns the comment string

    :   **Returns:**: the comment string

---


### toString

```
public java.lang.String toString()
```

:   returns the display string of the Type

    :   **Overrides:**: `toString` in class `java.lang.Enum<TechnicalInformation.Type>`
    :   **Returns:**: the display string


---


|  |  |  |
| --- | --- | --- |
| **Overview** \| **Package** \| **Class** \| **Tree** \| **Deprecated** \| **Index** \| **Help** \| **Weka's home** | |  |
| **PREV CLASS**   **NEXT CLASS** | **FRAMES**    **NO FRAMES**     **All Classes** |  |
| SUMMARY: NESTED \| ENUM CONSTANTS \| FIELD \| METHOD | DETAIL: ENUM CONSTANTS \| FIELD \| METHOD |  |


---
